# Une cause rare de cervicalgie febrile

**DOI:** 10.11604/pamj.2016.25.61.9315

**Published:** 2016-10-03

**Authors:** Zeineb Alaya, Walid Osman, Houneida Zaghouani, Nader Naouar, Chakib Kraiem, Elyès Bouajina

**Affiliations:** 1 Service de Rhumatologie, CHU Farhat Hached, Sousse, Tunisie; 2 Service de Chirurgie orthopédique, CHU Sahloul, Sousse, Tunisie; 3 Service d’Imagerie médicale, CHU Farhat Hached, Sousse, Tunisie

**Keywords:** Cervicalgie, fièvre, syndrome de la dent couronnée, Cervical pain, fever, cervical CT-scan, crowned dens syndrome

## Abstract

Une cervicalgie fébrile est souvent secondaire à une méningite ou à une spondylodiscite, exceptionnellement à une arthropathie microcristalline. Nous en rapportons un cas. Un homme de 81 ans sans antécédents particuliers est hospitalisé pour cervicalgie fébrile. Les diagnostics initialement évoqués étaient ceux de méningite et de spondylodiscite. L'examen a montré une raideur globale du rachis cervical. L'IRM rachidienne a montré une anomalie de signal de l'articulation atloïdo-axoïdienne se réhaussant après injection de gadolinium avec une hypertrophie synoviale associée à un aspect irrégulier et hétérogène de la dent de l'axis. Les coupes tomodensitométriques atlo-axoïdiennes montraient des calcifications péri-odontoïdales confirmant le diagnostic de syndrome de dent couronnée (SDC). L'évolution était favorable sous AINS. Le SDC mérite d'être mieux connu ; il peut mimer de nombreux diagnostics et être responsable de fièvre au long cours.

## Abstract (en)

Febrile cervical pain is often secondary to meningitis or spondylodiscitis and, exceptionally, to microcrystalline arthropathy. We here report a case. A 81-year old man with no particular personal history was hospitalized with febrile cervical pain. Initial diagnoses were meningitis and spondylodiscitis. Clinical examination showed overall stiffness of cervical rachis. Spinal MRI showed abnormality of the atlanto-axial articulation signal, enhanced after gadolinium injection with synovial hypertrophy associated with irregular and heterogeneous aspect of the dens of the axis. Serial atlanto-axial Computed Tomography (CT) scan showed peri-odontoid calcifications, confirming the diagnosis of crowned dens syndrome (CDS). Patient evolution was favorable under nonsteroidal anti-inflammatory drugs (NSAIDs). CDS deserves to be better known; it can mimic many disorders and be responsible for long term fever.

## Introduction

Le syndrome de la dent couronnée (SDC), appelé crowned dens syndrome par les Anglo-Saxons [1], correspond à une calcification du ligament transverse de l´atlas, réalisant une demi-couronne dense enserrant la partie postérieure de la dent de l´axis [2]. Ses manifestations cliniques peuvent être trompeuses [3]. Nous en rapportons un cas.

## Patient et observation

**Figure 1:**
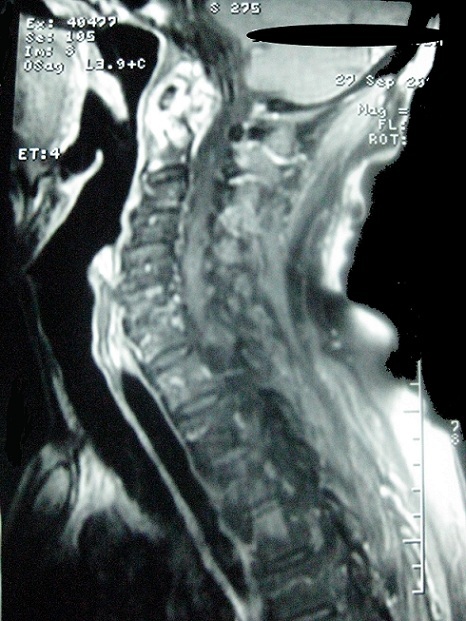
IRM rachidienne en coupe sagittale: anomalie de signal de l’articulation atloïdo-axoïdienne se réhaussant après injection de gadolinium avec hypertrophie synoviale associée à un aspect irrégulier et hétérogène de la dent de l’axis

**Figure 2:**
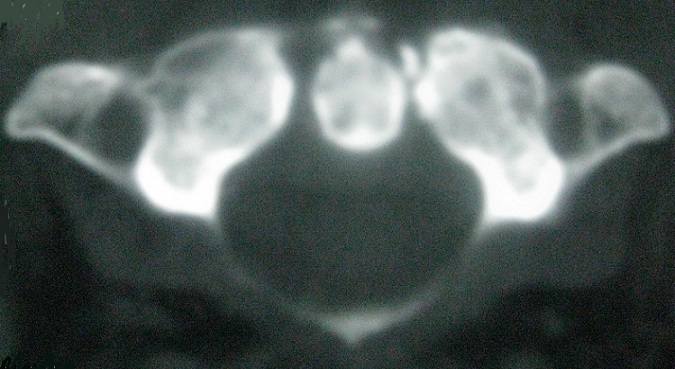
TDM du rachis cervical en coupe axiale: présence de fines calcifications arciformes qui coiffent la dent de l’axis avec aspect irrégulier de la dent confirmant de diagnostic de SDC

**Figure 3:**
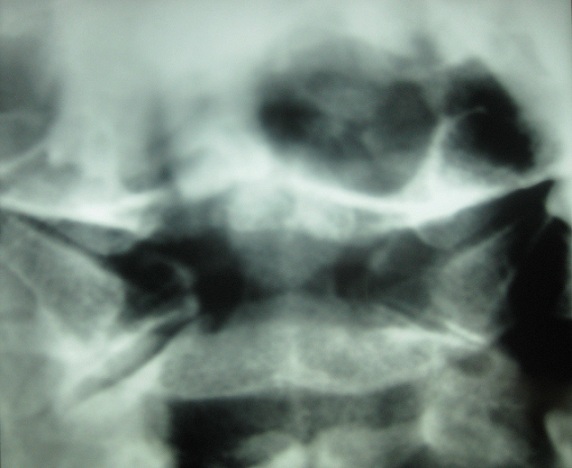
Radiographie du rachis cervical incidence C1-C2 (bouche ouverte): calcifications péri-odontoïdales

Un homme de 81 ans sans antécédents particuliers est hospitalisé pour des cervicalgies fébriles évoluant depuis 2 mois. À l'examen, le rachis cervical était raide dans toutes les amplitudes avec une fièvre à 38.2°C. L'examen neurologique était normal ainsi que le reste de l'examen clinique. Il existait une hyperleucocytose à 12 000/mm^3^avec polynucléose, une CRP à 76 mg/L et une VS à 85 mm à la première heure. Le bilan phosphocalcique était normal. Les diagnostics de méningite ou de spondylodiscite infectieuse étaient évoqués. Une ponction lombaire est alors effectuée en urgence avec une analyse du liquide céphalorachidien strictement normale. Les radiographies du rachis cervical ne mettaient pas en évidence d'anomalies particulières. L'IRM rachidienne a montré une anomalie de signal de l'articulation atloïdo-axoïdienne se réhaussant après injection de gadolinium avec une hypertrophie synoviale associée à un aspect irrégulier et hétérogène de la dent de l'axis (Figure 1). Les radiographies standard du thorax et des sinus, une panoramique dentaire ainsi que les prélèvements microbiologiques (hémocultures, ECBU) étaient normaux. L´examen endobuccal de même que la palpation des points sinusiens et des artères temporales et occipitale étaient normaux. Le bilan immunologique (FR, Anti-CCP et AAN) était négatif. Le diagnostic de SDC était alors évoqué et confirmé par des coupes tomodensitométriques atlo-axoïdiennes ayant montré des calcifications péri-odontoïdales (Figure 2). La radiographie du rachis cervical en incidence bouche ouverte « C1-C2 » a retrouvé ces calcifications péri-odontoïdiennes (Figure 3). Dans le but de préciser l´étiologie microcristalline du SDC, la réalisation de radiographies standard des épaules, du bassin, des genoux et des poignets a révélé un liséré calcique aux genoux et aux épaules évoquant une chondrocalcinose articulaire (CCA). Un traitement par AINS par voie orale (célécoxib) a permis d'obtenir la disparition des douleurs en 48 heures avec normalisation des paramètres biologiques inflammatoires.

## Discussion

Le SDC constitue une localisation particulière de la CCA ou moins fréquemment du rhumatisme à hydroxyapatite [2]. Ce syndrome touche surtout la femme avec un âge moyen de diagnostic compris entre 60 et 70 ans [2, 3]. Il s´agit donc d´un syndrome radio-clinique dont la définition radiologique s´est élargie, incluant toutes les images de calcifications des structures abarticulaires odonto-atloïdiennes identifiées au pourtour et/ou au dessus de l´apophyse odontoïde et se rapportant à ce même syndrome clinique. Dans les éléments diagnostiques du SDC, il faut insister sur la valeur fondamentale de l´interrogatoire : il est en effet fréquemment retrouvé des épisodes d´arthrite ou de tendinite aiguës calcifiantes aux poignets, aux épaules ou aux genoux, dans le cadre d´une CCA ou d´un rhumatisme à hydroxyapatite [2, 3]. Le SDC associe des douleurs cervicales aiguës ou chroniques et une diminution de la mobilité du rachis cervical dans toutes les directions [2, 3]. La présentation clinique du SDC peut être explosive et doit faire éliminer en premier lieu, notamment dans un contexte fébrile, une méningite ou une spondylodiscite infectieuse [3, 4]. En présence d´une cervicalgie fébrile, la découverte de calcifications évocatrices de SDC ne doit faire retenir ce diagnostic qu´après avoir éliminé de façon certaine ces deux derniers diagnostics [2–4]. L'IRM est surtout utile à l'élimination des diagnostics différentiels: méningite [4], ostéomyélite [5], spondylodiscite [4], arthrose [4], spondylarthrite ankylosante [6], goutte [7], polyarthrite rhumatoïde [4], métastase [4], tumeur rachidienne [4]. Elle permet, comme la scintigraphie osseuse, d'établir l'imputabilité des calcifications dans la symptomatologie en montrant, par la réalisation de séquence STIR, un hypersignal localisé à l'articulation atloïdo-axoïdienne [8]. Le scanner centré sur la dent odontoïdienne en fenêtre osseuse est l´examen de référence du diagnostic de SDC, permettant d´identifier ses diverses formes radiologiques : calcification en simple bande, en double et fin liséré le long du ligament rétro-odontoïdien, calcifications en mottes irrégulières, en couronne autour ou au dessus de la pointe de la dent odontoïdienne, pouvant conduire à son érosion [2, 9, 10]. Les radiographies standard centrées sur l´apophyse odontoïde, idéalement « l´incidence de Blondeau bouche ouverte », peuvent quelques fois montrer ces calcifications [2]. Le scanner peut lui-même être pris en défaut lorsqu´il est pratiqué tardivement et apparaître normal ou peu pathologique, car ces calcifications peuvent se résorber [11, 12]. Les radiographies articulaires peuvent par ailleurs aider à préciser l´étiologie (chondrocalcinose ou hydroxyapatite) du SDC [3]. Le traitement du SDC repose sur la prise d'anti-inflammatoires ou de la colchicine [3, 4]. Le SDC évolue favorablement sous traitement médical [3, 4] avec dans notre cas une disparition en 48h de la fièvre et des cervicalgies sous anti-inflammatoire non stéroïdien.

## Conclusion

Le SDC est un diagnostic rare et difficile. Les images radiologiques et l'absence d'arguments solides en faveur d'une atteinte infectieuse ou tumorale sont indispensables pour porter ce diagnostic.
